# Winter Air Pollution from Domestic Coal Fired Heating in Ulaanbaatar, Mongolia, Is Strongly Associated with a Major Seasonal Cyclic Decrease in Successful Fecundity

**DOI:** 10.3390/ijerph18052750

**Published:** 2021-03-09

**Authors:** Jargalsaikhan Badarch, James Harding, Emma Dickinson-Craig, Colleen Azen, Hilary Ong, Samantha Hunter, Pia S. Pannaraj, Brigitta Szepesi, Tegshjargal Sereenendorj, Sumiya Davaa, Chimedsuren Ochir, David Warburton, Carol Readhead

**Affiliations:** 1 Department of Obstetrics and Gynecology, Mongolian National University of Medical Sciences, Ulaanbaatar 14210, Mongolia; jargalsaikhan.b@mnums.edu.mn; 2 Urguu Maternity Hospital, Ulaanbaatar 14210, Mongolia; sumya_1997@yahoo.com; 3 The Saban Research Institute, Children’s Hospital Los Angeles, Keck School of Medicine, University of Southern California, Los Angeles, CA 90027, USA; hardingj@usc.edu (J.H.); CAzen@chla.usc.edu (C.A.); Hilary.Ong@ucsf.edu (H.O.); shunter@chla.usc.edu (S.H.); ppannaraj@chla.usc.edu (P.S.P.); 4 Institute for Applied Health Research, University of Birmingham, Birmingham B15 2TT, UK; exd852@student.bham.ac.uk; 5 United States Naval Academy, Annapolis, MD 21402, USA; themuse4x1@gmail.com; 6 Mongolian Association of Obstetrics Gynecology and Neonatology, P.O. Box 802, Ulaanbaatar 14210, Mongolia; tegshjargal.s99@gmail.com; 7 Ministry of Health, Ulaanbaatar 14210, Mongolia; chimedsuren@mnums.edu.mn; 8 Biology and Bioengineering, California Institute of Technology, Pasadena, CA 91125, USA; readhead@caltech.edu

**Keywords:** winter air pollution, conception, fecundity, Ulaanbaatar Mongolia

## Abstract

Pollution of the environment is increasing and threatens the health and wellbeing of adults and children around the globe. The impact of air pollution on pulmonary and cardiovascular disease has been well documented, but it also has a deleterious effect on reproductive health. Ulaanbaatar, the capital city of Mongolia, has one of the highest levels of air pollution in the world. During the extreme winters when temperatures routinely fall below −20 °C the level of air pollution can reach 80 times the WHO recommended safe levels. Heating mainly comes from coal, which is burned both in power stations, and in stoves in the traditional Ger housing. We studied the impact of air pollution on conception rates and birth outcomes in Ulaanbaatar using a retrospective analysis of health data collected from the Urguu Maternity hospital in Ulaanbaatar, Mongolia. Daily levels of SO_2_, NO_2_, PM_10_, and PM_2.5_ were collected from the government Air Quality Monitoring Stations in Ulaanbaatar for the same period as the study. In January, the month of highest pollution, there is a 3.2-fold decrease in conceptions that lead to the successfully delivered infants compared to October. The seasonal variations in conceptions resulting in live births in this study in Ulaanbaatar are shown to be 2.03 ± 0.20 (10-sigma) times greater than those in the Denmark/North America study of Wesselink et al., 2020. The two obvious differences between Ulaanbaatar and Europe/North America are pollution and temperature both of which are extreme in Ulaanbaatar. The extreme low temperature is mitigated by burning coal, which is the main source of domestic heat especially in the ger districts. This drives the level of pollution so the two are inextricably linked. Infants conceived in the months of June-October had the greatest cumulative PM_2.5_ pollution exposure over total gestation, yet these were also the pregnancies with the lowest PM_2.5_ exposure for the month of conception and three months prior to conception. The delivered-infant conception rate shows a markedly negative association with exposure to PM_2.5_ prior to and during the first month of pregnancy. This overall reduction in fecundity of the population of Ulaanbaatar is therefore a preventable health risk. It is of great consequence that the air pollution in Ulaanbaatar affects health over an entire lifespan including reproductive health. This could be remedied with a clean source of heating.

## 1. Introduction

Ambient air pollution is a global environmental issue that affects human health [1,2,3], and there can be rapid and substantial benefits from reducing pollution at its source [4]. Worldwide, over 4.9 million deaths in 2017 have been attributed to air pollution [2]. Air pollution is primarily associated with overall mortality and with mortality due to cardiovascular and respiratory diseases [5,6]. Studies conducted over a wide geographical area including Asia, have shown that long-term exposure to ultra-fine carbon based particles of PM_2.5_ increases the risk of mortality from cardiovascular disease (CVD) [4]. Air pollution can also damage other organ systems [6] and is linked to malignancies such as bladder cancer [7], and childhood leukemia [8]. Mongolian children exposed to air pollution have poorer lung development and have a higher prevalence of asthma [9,10]. Air pollution also has a deleterious effect on reproductive health, including the growth and development of the fetus, low birth weight, and preterm births [11,12]. In Ulaanbaatar winter air pollution is also strongly correlated with spontaneous abortion [13]. Our previous studies have focused on the impact of air pollution on the health of the people of Ulaanbaatar, Mongolia across their lifespan [13,14,15]. In this study we focus on the association of fecundity with pollution.

Ulaanbaatar (UB), the capital city of Mongolia, is considered the coldest capital in the world with winter temperatures often dropping below −20 °C. Ulaanbaatar has an approximate population of 1.5 million people, which is close to half the population of Mongolia (Mongolian Statistical Information Service), and around 60% of UB’s population live in traditional housing called gers [16,17,18]. In the gers, small central stoves in the main living and sleeping room are used for cooking and heating, and during the winter each household burns 3 to 6 tons of raw coal [19]. Ulaanbaatar has 4 coal burning power stations, which serve the city to provide heating via steam ducts, but these contribute <15% of the overall air pollution. The burning of coal in domestic stoves is the major source of air pollution in Ulaanbaatar in the winter months, but vehicles also contribute to pollution year round. As Ulaanbaatar lies in a valley surrounded by mountains, an inversion layer prevents the pollution from being dissipated to the open spaces beyond the city [18]. In winter all classes of particulate matter increase, with UB having the second highest PM_10_ concentrations globally [19]. Particulate matter can reach levels many times the World Health Organization (WHO) recommended safe levels [2]. For instance, in December and January of 2016, peak PM_2.5_ particulate matter levels of more than 2000 µg /m^3^ were recorded by the US Embassy adjacent to the northern Ger district. This PM_2.5_ level is 80 times the WHO recommended safe daily average of 25 µg/m^3^ of PM_2.5_ [19]. Atmospheric, gaseous and particulate matter pollution poses an urgent public health threat to the city’s inhabitants over their lifespan [15]. 

Infertility in humans has continued to increase in developed countries [20,21]. There are many factors that have an impact on this emerging complex issue including: underlying health conditions such as obesity [22], socio-economic factors [23], environmental toxins [24], chromosome disorders of the embryo [25], and seasonal variations [2,26]. However, there is mounting evidence that air pollution is deleterious to human reproductive health and fecundity [3,4]. Particulate matter, particularly PM_2.5_, has been associated with poor pregnancy outcomes [26].

We have reported previously that in Ulaanbaatar high levels of ambient pollutants correlate strongly with spontaneous abortion [13]. In our present study we aimed to analyze whether there is an association between ambient pollution levels and overall fertility and fecundity in the calendar months during which infants born at Urguu Maternity Hospital (UMH) in Ulaanbaatar were conceived. By doing this, we aimed to challenge the null hypothesis that ambient pollution levels in UB have no adverse effects on overall conception and fecundity of the population. 

## 2. Materials and Methods

The medical records of 10,715 women residing within UB city limits and admitted for delivery to the UMH between January 2014 and December 2015 were collected, de-identified and examined retrospectively. Data collected from medical records included maternal age, gestational age (GA) in weeks and pregnancy outcome. Spontaneous abortion, stillbirths or the presence of any serious maternal medical conditions were a predefined exclusion criterion. All delivery events including vaginal and caesarian deliveries were included in the analysis. The year and month of conception for each birth was derived by subtracting gestational age (GA) at delivery in weeks from the date of birth, which is referred to as the Delivered-Infant Conception Date (DICD).

Monthly averaged daily levels of SO_2_, NO_2_, PM_10_, and PM_2.5_ between March 2013 and December 2015 from the 6 Government of Mongolia Air Quality Monitoring Stations within UB city limits were obtained. The extreme winter weather in UB drives coal burning and thus pollution each year. This has been a consistent pattern for the past decade.

Statistical analysis was done using Prism GraphPad 8 (8.4.3) to determine correlation between monthly conceptions and air pollutants by means of regressions analyses, expressed as r coefficients. A significance of fit was accepted for correlations at the *p* < 0.05 level, although the key anti-correlations presented here are significant at much higher levels (*p* < 0.05). 

The ratio of natural seasonal variation in pregnancy initiation attempts to average over 12 months from Wesselink et al., 2020 [1] was compared to the ratio of conceptions to average over 12 months in our data. We followed the Wesselink et al., 2020 [1] approach in fitting our data to a sine curve with a twelve month period.

Ethics approval and consent to participate was granted by the IRB at UMH, and the Mongolian National University of Medical Sciences (MNUMS), Ulaanbaatar, Mongolia. This paper describes a NIH IRB type 4 exemption de-identified human subjects project.

Data and materials are held by Dr. Jargalsaikhan at UMH and MNUMS, and by Dr Warburton at the Saban Research Institute. The datasets used and/or analyzed during the current study are available from the corresponding author on reasonable request. The authors have no competing interests.

## 3. Results

### 3.1. Birth Data

A total of 10,715 live births were delivered vaginally at UMH between January 2014 and December 2015, of which the majority were delivered at full term (Table 1).

The number of deliveries by cesarean section averaged 434 per month and did not vary significantly with the seasons. Inclusion or exclusion of caesarian births did not affect the statistical validity of the conclusions.

**Figure 1:**
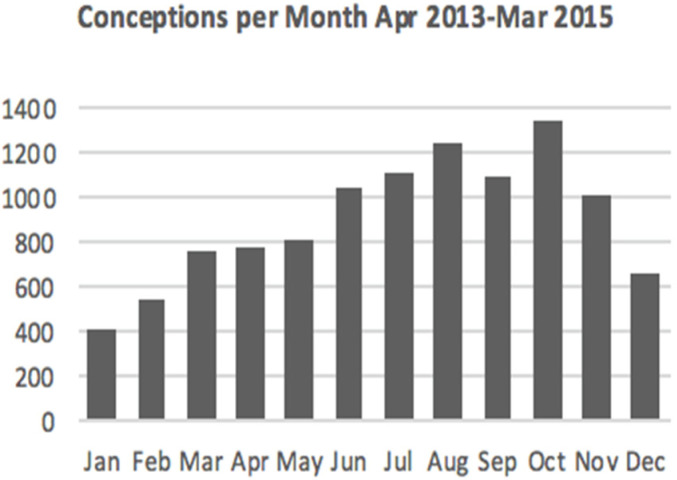
Shows the Delivered-Infant Conception Dates (DICD) of the infants that were delivered between January 2014 and December 2015 (Table 1). The conception date was deducted from the gestational age at birth.

The raw data showed clearly that monthly births by vaginal delivery had a seasonal variability, with autumn months having the lowest frequency, averaging 582 births per month between September and December. Delivered-Infant Conception Date (DICD), deducted from the gestational age (GA) at birth, varied by season (Figure 1). In particular, there was a 3.2-fold decrease in the number of successfully delivered infants that were conceived in January compared with October.

### 3.2. Conception, Spontaneous Abortion, Winter Temperatures and Air Pollution

**Figure 2:**
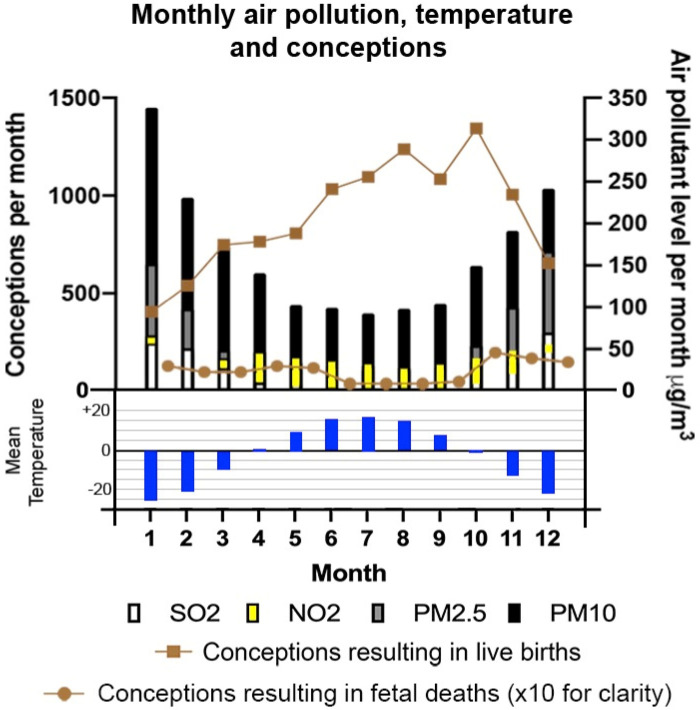
Monthly successful conception rates (brown line with squares) versus the mean total monthly air pollution concentrations with SO_2_ (white bars), NO_2_ (yellow bars), PM_2.5_ (gray bars), and PM_10_(black bars). Monthly conception rates that resulted in early (<20 weeks) fetal death (brown line with circles). The latter data has been taken from our previous study in 2011 by Enkhmaa et al., normalized [13] and multiplied by 10 for clarity. The mean monthly temperature is plotted below (blue bars).

Comparison of birth rates versus the mean monthly air pollution concentrations for all measured pollutants showed that high daily pollution levels during the month of conception were associated with significantly fewer infants being born successfully at the hospital (Figure 2, *p* < 0.05). Low temperatures drive the burning of coal for warmth and are therefore inextricably linked to high pollution levels (Figure 2).

**Figure 3:**
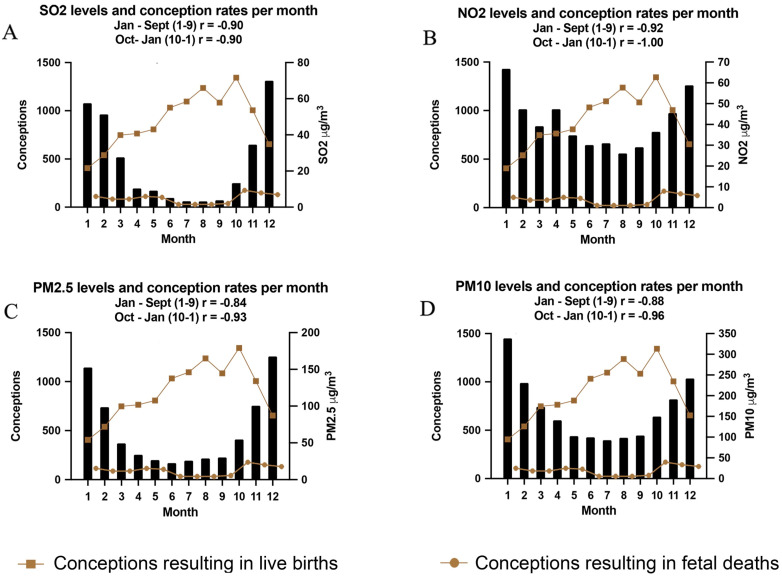
Monthly average levels of daily air pollutants SO_2_, NO_2_, PM_2.5_ and PM_10_ (**A**–**D**, respectively) and successful conceptions per month at UMH from April 2013–March 2015. Correlations between each pollutant level SO_2_, NO_2_, PM_2.5_ and PM_10_ and successful conceptions were calculated from January to September, and from October to January. Monthly conception rates that resulted in early (<20 weeks) fetal death (brown line with circles) are shown. This data is from our previous study by Enkhmaa et al. [13]. These data points have been multiplied by 10 for clarity.

Figure 3 shows that the Delivered-Infant Conception Date (DICD) calculated number of conceptions per month was negatively correlated with low winter temperatures and high air pollution for each pollutant measured. NO_2_ had the strongest negative correlation January to September (1–9) r = −0.92 and October to January (10–1) r = −1.0, while SO_2_ had value r = –0.90 January to September (1–9) and r = −0.90 October to January (10–1). PM_2.5_ had a negative correlation of r = −0.84 from January to September (1–9) and r = −0.93 from October to January. PM_10_ r = −0.88 from January to September (1–9) and r = −0.96 from October to January (10–1). Each of the pollutants measured: SO_2_, NO_2_, PM_2.5_ and PM_10_ were negatively correlated with successful conceptions (*p* < 0.05) (Figure 3A–D). A significant increase in early spontaneous abortion of fetuses that was associated with conceptions during the months of high pollution has been reported previously [13]. The number of early spontaneous abortions during months of high pollution is significant although small compared to the decrease in successful conceptions during the same months

### 3.3. Conception and SO_2_ Pollution

**Figure 4:**
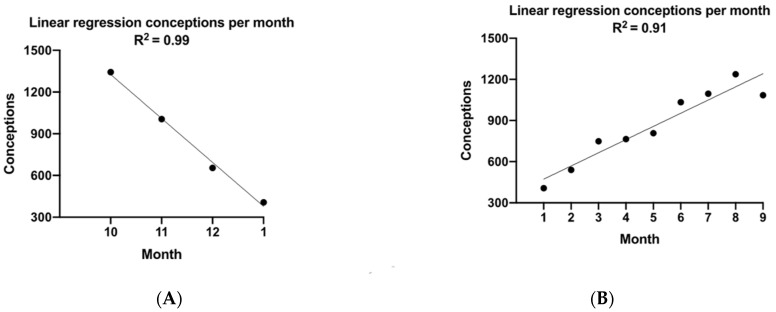
(**A**): Dose–response model for SO_2_ on conceptions; the winter rise in atmospheric SO_2_ begins in October, then peaks in January, while the corresponding monthly DICD falls linearly with the rise in SO_2_ levels (R^2^= 0.99, *p* < 0.05). (**B**): Elimination Kinetics Model. Following the polluted winter months, atmospheric SO_2_ drops to low levels by March (see Figure 3A), but the rise in conceptions (DICD) lags this steep fall in SO_2_ but then recovers linearly with time (R^2^ = 0.91, *p* < 0.05), suggesting that the negative effect of SO_2_ on this population’s DICD washes out with zero-order kinetics over the summer, but returns rapidly with the onset of the following winter time pollution.

As monthly mean atmospheric concentrations of SO_2_ rose from October to January, we found a corresponding rapid decline in the DICD calculated conceptions, which could be closely modeled as a linear dose–response curve using linear regression (R^2^= 0.99) with significant fit (*p* < 0.05) (Figure 4A). Yet, as SO_2_ concentrations returned to lower levels in the spring and summer months (April–September, Figure 3A), there was not an immediate return to peak DICD calculated conception numbers, rather DICD was found to recover linearly yet relatively slowly over the summer, R^2^ = 0.91 (Figure 4B), suggesting that the inhibitory effect of SO_2_ on this population’s conception rate slowly decreases with zero-order kinetics. 

### 3.4. Conception and PM_2.5_ Exposure

**Figure 5:**
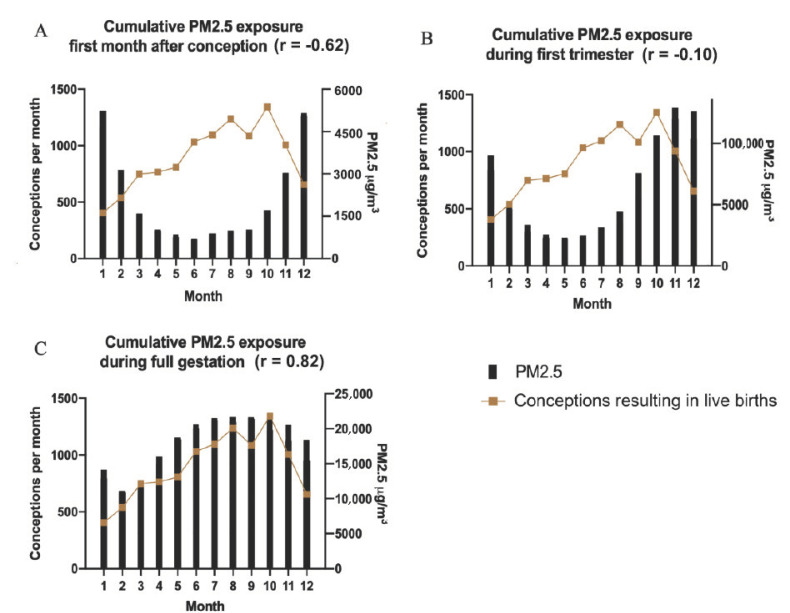
Cumulative daily maternal exposure to PM_2.5_ during the first month of pregnancy (**A**), the first trimester (**B**) and full gestation (**C**).

PM_2.5_ particles breathed into the lung can enter the blood stream where they can be carried to all the organs of the body. Epidemiological studies of humans and animal experiments have shown maternal exposure to PM_2.5_ results in abnormal placenta development [27]. Gestational exposure of mice to PM_2.5_ impairs vascularization of the placenta [28]. It also results in preterm birth and low birth weights [29]. We have shown that in UB there is a correlation between PM_2.5_ exposure and spontaneous abortions [13]. Since PM_2.5_ levels differ greatly between summer and winter in UB, we were able to investigate whether certain stages of pregnancy were more vulnerable to PM_2.5_ exposure than others. The cumulative daily exposure to PM_2.5_ during the stages of pregnancy is shown in Figure 5: the first month of pregnancy Figure 5A; the first trimester Figure 5B; and the entire pregnancy Figure 5C. It is an interesting fact that the DICD was negatively correlated (r= −0.62; *p* < 0.05) with cumulative exposure to PM_2.5_ pollution during the first month of pregnancy (Figure 5A), but there was no significant negative correlation of DICD with cumulative PM_2.5_ exposure over the first trimester or over total gestation (Figure 5B,C, respectively). In fact, infants conceived in the months of June-October, had the greatest cumulative maternal PM_2.5_ pollution exposure over total gestation, but these were also the pregnancies with the lowest PM_2.5_ exposure both prior to conception and during the first month of gestation (Figure 5A). The month preceding fertilization and the first month of pregnancy show the strongest negative association between conceptions leading to live births and pollution, while there is little indication of a negative association between high exposure to pollution during the remaining 8 months of pregnancy (Figure 5C). 

### 3.5. Seasonal Patterns in Successful Conceptions in UB Compared with Those in North America and Denmark

**Figure 6:**
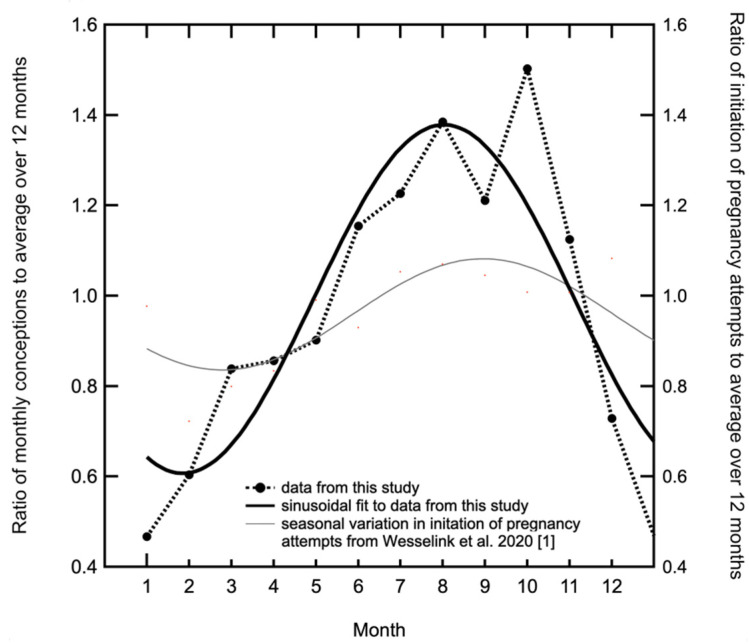
The monthly ratio to the annual mean of the conceptions resulting in live births in the present study. The heavy black curve is a sinusoidal least square fit to the data constrained to have a period of one year. The amplitude is 38.6% ± 5.9% of the mean monthly conceptions. Seasonal patterns in pregnancy initiation attempts from two cohorts PRESTO (5827 women) from Canada and North America and Snart Gravid (8504 women) from Denmark [1] (data from a total of 14,331 women), were combined to form the weighted average and fitted with a sinusoid constrained to have a period of one year, which is shown by the thin black curve, which has an amplitude of 12.3% ± 3.0%.

Wesselink et al., 2020 [1] accounted for the seasonal variation in initiation of pregnancy attempts and then showed a residual modest seasonal variation in fecundability with a peak in the late fall and early winter in both cohorts. They found a peak to low ratio in North America of 1.16 (95% confidence interval: 1.05, 1.28) and in Denmark of 1.08 (95% confidence interval: 1.00, 1.16). The seasonal variation in initiation of pregnancy attempts to their combined cohorts has been fitted by the annual sinusoidal curve shown by the thin line in Figure 6 which has amplitude 12.3% ± 2.1%.

Since we do not have any data on the initiation of pregnancy attempts in UB we are here taking the results from North American and Denmark [1] as a proxy for UB.

Humans and some species of nonhuman primates, in contrast to most mammals, are not considered seasonal breeders [30]. However, it has been widely observed and documented that there are seasons of greater fecundity [31,32], which as we have seen (Figure 6) [1] are small. 

A fitted annual sinusoid curve to our data is shown in Figure 6. This has amplitude 38.6% ± 5.9% of the mean monthly conceptions. The peak to low ratio for our data is 2.27 (95% confidence interval: 1.85, 2.70). Combining the cohorts in the Wesselink et al., 2020 [1] data we find a peak to low ratio of 1.12 (95% confidence interval: 1.05, 1.19). There is a highly significant difference between the UB data and those from North America plus Denmark. The ratio of the peak to low in our study compared to that in the Wesselink et al., 2020 [1] study is 2.03 ± 0.20, i.e., a 10-sigma difference that must be ascribed to different conditions between the two population groups. The much more dramatic changes in conception rates in Ulaanbaatar are clearly associated with high levels of winter pollution.

## 4. Discussion

The goal of this study was to determine whether high levels of pollution from the burning of coal during the winters in Ulaanbaatar had any impact on the conception and successful delivery of infants. To do so, we devised the Delivered-Infant Conception Date (DICD) to express the monthly conception rates of infants presenting for successful delivery at UMH. This novel DICD statistic could be measuring aspects of fecundity such as fertility rate or spontaneous early abortion rate. We have shown previously that the rate of spontaneous early fetal loss at <20 weeks increases significantly up to 4-fold in a strong correlation with levels of ambient pollution during the winter months in UB, but falls rapidly during the early summer months with decreasing levels of several air pollutants, including those studied here [13]. Therefore, the finding that fewer infants were apparently conceived in the high pollution winter months may be due not only to a lower rate of conception (i.e., fertility), but may also be contributed to by an increase in the rate of miscarriages. However, in our previous study, the rate of fetal loss or miscarriage of established pregnancies was relatively small as can be seen in Figure 2 and Figure 3 and could not account for the decline in conceptions calculated here using DICD as we showed by comparing the data (note that in Figure 2 and Figure 3 the scale for the spontaneous abortions has been multiplied by a factor of 10 for clarity). Thus, an increase in the rate of spontaneous abortion, especially during the first trimester of pregnancy could only partially explain some 7% of the herein reported variation in DICD. Natural seasonal variation in human conception [1] was likewise modest compared to the three-fold decrease in conception in the polluted winter months in UB. 

The highest number of successful conceptions (DICD) occurred in the early fall, yet these pregnancies incurred the highest accumulated exposure to pollution overall, as shown in Figure 5C. It is well known that implantation and early postimplantation development are the most sensitive and vulnerable stages of pregnancy and some women suffer from repeated implantation failure (RIF) [33]. In spite of extensive efforts, IVF clinics have not been able to improve implantation substantially [34,35]. 

Moreover, importantly, the recovery curves for early fetal loss and DICD are clearly distinct and different. Unlike the recovery pattern of spontaneous abortion, which is closely correlated with rapidly falling pollution levels in the spring and early summer, we found a marked lag in the recovery of conception measured as DICD following the end of the high-pollution season. This is seen clearly in Figure 2, where the maximum conception leading to live births occurs in the late summer/early fall while the pollution has already dropped to its summer plateau by May. This is a finding, which we interpret as suggesting a more durable adverse physiological effect of pollution on overall fecundity, consistent with an inhibitory impact of pollutants on the initiation of new pregnancies. Moreover, the recovery lag phase appears to clear by first order linear kinetics over the summer. This effect is rapidly reinitiated with the onset of the next round of winter pollution.

Thus the prolonged, yet eventually linearly reversible, impact of pollutants on this large Mongolian population’s fecundity suggests that seasonal high winter pollutant concentrations are strongly correlated with a seasonal, negatively correlated, dose–response effect that is inhibiting fertility and thus overall fecundity. One possible explanation for this finding could be that the extreme levels of air pollution seen during UB winter months are leading to a marked yet slowly recoverable inhibition of spermatogenesis or perhaps implantation, which is not immediately resolved when the levels of gaseous and particulate matter by-products from coal burning drop to near WHO safety guidelines in late spring. High levels of air pollution including PM_2.5_ and SO_2_ have been shown to have a negative impact on spermatogenesis [36,37]. If maturation of spermatozoa progenitors were impaired in this manner, we might expect there to be such a lag in the recovery of fecundity (Figure 2) after the pollution dose falls to summer background levels, because maturation of spermatozoa from spermatogenic stem cells takes ∼30–40 days [38,39,40]. This hypothesis would be amenable to field-testing in future studies and should perhaps be examined in other global populations exposed to high pollution levels.

## 5. Conclusions

There were 3-fold fewer infants born vaginally at UMH when the month of conception fell during the heavily polluted winter months. There is therefore an alarming linear negative correlation between successful completion of pregnancy and high levels of winter air pollution at the time of conception. This apparently strongly associated dose–response curve recovered gradually over the summer with first order kinetics, suggesting a slow pollution wash out that we speculate may reflect delayed recovery of spermatogenesis or perhaps of implantation, or both. Thus, both the lower conception and therefore eventual birth rate and the heavy winter coal smoke pollution must be considered to be strongly correlated, indicating potentially preventable health risks to the overall fecundity of the Mongolian population living in UB. 

## Figures and Tables

**Table 1:** Shows the gestational age of the infants that were delivered between 14 January 2014 and December 2015.

Gestational Age (weeks)	Number of Births (Male/ Female)	Percentage of Total
≤35	158 (64/94)	1
36	180 (71/109)	2
37	394 (218/176)	4
38	989 (528/461)	9
39	2383 (1303/1080)	22
40	4042 (2069/1973)	38
41	2569 (1214/1355)	24
Total	10715	100

## Data Availability

The data presented in this study are available on request from the corresponding author. The data are not publicly available.
